# 

**Published:** 1922-09

**Authors:** 


*The following donations have been received since the publication of the List in December,* 1921.

*30th September,* 1922.

| | | |
|---|---|---|
| Mr. A. L. Flemming (1) | .. .. .. | 1 volume. |
| Dr. R. G. Poole Lansdown (2) | .. .. | 1 ,, |
| Medical Research Council (3) | .. .. .. | 2 volumes. |
| Mr. R. H. Norgate (4) | .. .. .. .. | 1 volume. |
| Dr. J. E. Shaw (5) | .. .. .. .. | 1 ,, |
| Dr. Alexander Smith (6) | .. .. .. | 1 ,, |
| Dr. James Swain (7) | .. .. .. .. | 4 volumes. |
| Mr. J. Taylor (8) | .. .. .. .. | 2 ,, |

Framed portraits have been presented by Dr. Poole Lansdown and Dr. G. Parker. Unbound periodicals have been received from Mr. Hey Groves and Dr. Symes.

### THE ONE HUNDRED AND SIXTH LIST OF BOOKS.

The figures in round brackets refer to the figures after the names of the donors and show by whom the volumes were presented. The books to which no such figures are attached have either been bought from the Library Fund or received through the *Journal.*

**Arderne, J.** .. .. *De Arte Phisicali et de Cirurgia* (*Transcript, d.* 1412) (Trans. by Sir D'A. Power) .. .. 1922

**Ashby and Wright** .. *Diseases of Children* (Ed. by H. T. Ashby and C. Roberts) .. .. .. .. .. .. 6th Ed. 1922

**Bainbridge and J. A. Menzies, F. A.** *Essentials of Physiology* 4th Ed. 1920

**Ballance, Sir C. A.** *History of the Surgery of the Brain* .. .. 1922

**Bardswell, N. E.** [Ed.] *The Tuberculosis Clinic* .. .. .. .. 1922

**Bernheim, B. M.** .. *Blood Transfusion* .. .. .. .. .. .. (1) [1917]

**Bianchi, L.** .. .. *The Mechanism of the Brain* (Trans. by J. H. Macdonald) .. .. .. .. .. .. .. .. .. .. 1922

**Brand, A. T.** .. *Cancer : its cause, treatment and prevention* .. 1922

**Bryce, A.** .. .. *Intestinal Toxæmia* .. .. .. .. .. .. (8) [1920]

**Burrell and A. S. Macnalty, L. S. T.** *Report on Artificial Pneumothorax* .. .. .. .. .. .. .. .. .. .. .. (3) 1922

**Clarkson, E. R. T.** [Ed.] *The Venereal Clinic* .. .. .. .. .. 1922

**Cobb, I. G.** .. .. *Aids to Organotherapy* .. .. .. .. .. .. 1922

**Core, D. E.** .. .. *Functional Nervous Disorders* .. .. .. 1922

**Crocket, J.** .. .. *Physical Examination of the Chest* .. .. 1922

**Demaye, H.** .. .. *Le Médecin devant l'Assistance et l'Enseignement Psychiatriques* .. .. .. .. .. .. 1922

**Groves, E. W. H.** .. *Synopsis of Surgery* .. .. .. .. .. 6th Ed. 1922

**Haneborg, A. O.** .. *Effects of Alcohol upon Digestion* .. .. .. 1921

**Harvey, W.** .. .. *Anatomical Exercitationes concerning Generation* .. .. .. .. .. .. .. .. .. .. (4) 1653

**Hayes, R.** .. .. *Aachen Treatment of Syphilis* .. 4th Ed. 1922

**Hepburn, M. L.** .. *The Ophthalmology of General Practice* .. 1922

**Hilton-Simpson, M. W.** *Arab Medicine and Surgery* .. .. .. .. 1922

**Hutchison and J. Sherren, R.** *Index of Treatment* .. .. 8th Ed. 1921

**Ker, C. B.** .. .. *Manual of Fevers* .. .. .. [2nd Ed.] [1922]

**Kerr, J. M. M.** .. *Clinical and Operative Gynæcology* .. .. 1922

**Keynes, G.** .. .. *Blood Transfusion* .. .. .. .. .. .. .. [1922]

**Laurens, G.** .. .. *Oto-Rhino-Laryngology* (Trans. by H. C. Fox) 2nd Ed. 1922

**Le Clerc, D.** .. .. *The History of Physick* (Trans. by Drake and Baden) .. .. .. .. .. .. .. .. .. (5) 1699

**Little, E. M.** .. .. *Artificial Limbs* .. .. .. .. .. .. .. .. 1922

**Luke, T. D.** .. .. *Manual of Physio-Therapeutics* ..New Ed. 1922

**Macnamara, C.** .. *Diseases of the Eye* (Trans. into Bengalese by L. M. Mookerjee) .. .. .. .. .. (7) 1885

**Moore, I.** .. .. *Intrinsic Cancer of Larynx* .. .. .. .. .. 1921

*Post- Graduate Lectures delivered before the Fellowship of Medicine* .. 1922

**Pybus, F. C.** .. .. *Surgical Diseases of Children* .. .. .. .. .. 1922

*Radium, Medical Uses of* .. .. .. .. .. .. .. .. .. .. .. (3) 1922

**Reuterwell, O. P.** .. *Über die Elasticität der Gefasswande* .. .. 1921

**Sainsbury, H.** .. *The Heart as a Power Chamber* .. .. .. [1922]

**Shaw, H. B.** .. .. *Hyperpiesia and Hyperpiesis* .. .. .. .. [1922]

**Somerville, H.** .. *Practical Psycho-analysis* .. .. .. .. .. .. 1922

**Steadman, F. St. J.** *Bacteriology for Dental Students* .. .. .. 1922

**Stiles and M. F. Forrester-Brown, Sir H. J.** *Injuries of the Peripheral Spinal Nerves* .. .. .. .. .. .. .. .. 1922

**Sutton, Sir J. B.** .. *Tumours* .. .. .. .. .. .. .. .. 7th Ed. 1922

**Sydenham, T.** .. *Selected Works* (Ed. by J. D. Comrie) .. .. 1922

**Thompson, C. J. S.** *A Compendium of Pharmacopœias* .. 6th Ed. 1922

**Tidy, H. L.** .. .. *A Synopsis of Medicine* .. .. .. 2nd Ed. 1922

**Tubby, A. H.** .. .. *A Consulting Surgeon in the Near East* (2) [1920]

**Turrell, W. J.** .. *Electrotherapy* .. .. .. .. .. .. .. .. .. [1922]

**Verdon, W.** .. .. *Angina Pectoris* .. .. .. .. .. .. (8) 1920

**Voltz, F.** .. .. .. *Dosage Tables for Deep-therapy* .. .. .. 1922

**Walker, N.** .. .. *Introduction to Dermatology* .. .. 7th Ed. 1922

**Weaver, G. H.** [*et al*] *Practical Medicine Series*, Vol. I. .. .. .. 1922

**Ziembusch, L. V.** .. *Atlas of Syphilis* .. .. .. .. .. .. .. 1922

TRANSACTIONS, REPORTS, JOURNALS, ETC.

American Association of Genito-Urinary Surgeons, Transactions of Vol. XIV. 1921
American Laryngological, Rhinological and Otological Society, Transactions of .. .. .. .. .. .. .. .. .. .. .. .. 1921
American Ophthalmological Society, Transactions of Vol. XIX. 1921
American Pediatric Society, Transactions of the Vols. XXXIII., XXXIV. 1921–22
American Surgical Association, Transactions of the Vols. XXXVIII., XXXIX. 1920–21
Association of American Physicians, Transactions of the Vol. XXXVI. 1921
British Medical Journal, The.. .. .. .. Vol. II., 1921 ; Vol. I., 1922
College of Physicians of Philadelphia, Transactions of the Vol. XLII. 1920
Edinburgh Obstetrical Society, Transactions of the Vol. XLI. 1922
Hospitals—
 Episcopal Hospital Reports .. .. .. .. .. .. .. Vol. V. 1920
 St. Bartholomew's Hospital Reports .. .. .. Vol. LIV. 1922
International (17th) Medical Congress—Proceedings of the Section of History of Medicine .. .. .. .. .. .. .. .. .. .. (6) 1914
Do., Section of Surgery. 2 vols. .. .. .. .. .. .. .. (7) 1913
Lancet, The .. .. .. .. .. .. .. .. .. Vol. II., 1921 ; Vol. I., 1922
London Dermatological Society, Transactions of the .. .. .. 1921
Medical Annual, The .. .. .. .. .. .. .. .. .. .. .. .. .. 1922
Medical Society's Transactions .. .. .. .. .. .. Vol. XLIV. 1921
Ophthalmological Society of the United Kingdom, Transactions of the .. .. .. .. .. .. .. .. .. .. ..Vols. XL., XLI. 1920–21
Royal Society of Medicine, Proceedings of.. .. .. Vol. XIV. 1921
St. Andrew's Institute for Clinical Research, Reports of the Vol. I. [1922]
Smithsonian Reports for 1919 and 1920 .. .. .. .. .. 1921–22

### Obituary.

### Local Medical Notes.

At the Annual Meeting of the Bristol Division of the British Medical Association on July 7th, 1922, the following were elected :—*Chairman*, H. L. Ormerod, M.D. ; *Vice-Chairman*, L. A. Moore, Esq. ; *Hon. Treasurer*, G. Parker, M.D. D. Robertson, M.B., to the Executive Committee. *Hon. Sec.*, Duncan Wood, F.R.C.S.

UNIVERSITY OF BRISTOL, EXAMINATION RESULTS.

M.B., CH.B. BRISTOL.—J. R. Duerden. *Final Examination, Part I.* (*including Forensic Medicine*) : G. R. Dunn, I. Williams. (*Part I.*) : M. V. Joscelyne, D. Staley, F. K. Wilson. *Second Examination, Part I.:* T. A. Barnabas. *Second Examination, Part II. :* D. H. Beatson, R. V. Cooke, A. S. Cox, F. J. Farr, C. F. Hayhurst, C. F. R. Killick, A. C. Lysaght, W. C. Morris. H. E. C. Bentley, R. E. Satchwell.

D.P.H. BRISTOL.—J. B. Adams, E. D. D. Davies, B. A. Astley-Weston. *Part I. :* F. P. Mackie.

M.B., B.S. LOND.—H. J. McCurrich. *Group I. :* N. E. Kemm. *Intermediate Examination :* F. G. Mogg.

DIPLOMA IN DENTAL SURGERY.—*Second Examination:* R. G. Butterworth, G. J. Kerry, F. C. G. Lewis. *Third Examination:* J. J. G. Bishop, A. V. Harding, T. D. Lewis. *Final Examination :* K. A. Hunt, G. A. S. Thomas.

CONJOINT EXAMINING BOARD.—*Anatomy and Physiology :* F. Kemm, Mary E. G. Wilkinson. *Medicine :* F. J. Hector, H. L. Shepherd, D. Walker, B. A. Crook, J. M. Evans. J. A. Roberts. *Surgery:* F. H. Bodman,* A. J. Keevil,* H. L. Shepherd,* Doreen G. Walker,* E. C. Kenderdine, F. J. Hector.* *Midwifery :* F. J. Hector, H. L. Shepherd, J. A. Roberts, D. Walker, H. J. Spreadbury, J. M. Evans, C. H. Osmond.

L.D.S., R.C.S.—F. H. Woolley.

* Qualified.

